# Inactivation of *BAP1* and the Hippo Pathway Characterizes the Genomic Alterations of Peritoneal Mesothelioma

**DOI:** 10.3390/life16030385

**Published:** 2026-02-28

**Authors:** Maya Samuels, Madi Williams, Angela Hasan, Susan Rafie, Grace S. Saglimbeni, Beau Hsia, Sunil Nair, Sweety Aeilias, Abubakar Tauseef

**Affiliations:** 1 Santa Clara University, College of Arts and Sciences, Santa Clara, CA 95053, USA; msamuels@scu.edu (M.S.); mrwilliams@scu.edu (M.W.); ahasan2@scu.edu (A.H.); 2 Arizona State University West, Glendale, AZ 85306, USA; srafie234@gmail.com; 3 School of Medicine, Creighton University, Phoenix, AZ 85012, USA; gss37749@creighton.edu; 4 Internal Medicine Department, School of Medicine, Creighton University, Omaha, NE 68178, USA; sunilnair@creighton.edu (S.N.); abubakartauseef@creighton.edu (A.T.); 5 CHI (Catholic Health Initiatives) Health Immanuel, CommonSpirit Health, Omaha, NE 68122, USA; sweetyaeilias@creighton.edu

**Keywords:** peritoneal mesothelioma, AACR Project GENIE, somatic mutations, *BAP1*, *NF2*, *TP53*, p53, hippo signaling pathway, tumor suppressor genes

## Abstract

Background/Objectives: Peritoneal mesothelioma is a rare malignancy characterized by limited therapeutic options and a poor prognosis. Genomic characterization can enhance the understanding of the molecular mechanisms that lead to this disease and can contribute to improved survival outcomes through therapeutic targets. Methods: Analysis was performed using a dataset from the AACR GENIE database (v17.0-public) comprising 204 samples from 192 patients. Data were analyzed to identify patterns in genomic alterations and clinical demographics. Within the GENIE cohort, histologic subtype information was incomplete and inconsistently reported across contributing institutions. Hence, histological subtype genomic analysis was not viable. Results: The most common somatic mutation was found in the *BAP1* gene (25.98%). Other common mutations were found in the *NF2* (15.19%), *TP53* (9.3%) and *SETD2* (8.3%) genes. Several pathways were found as potential treatment targets including the chromatin remodeling, Hippo, and p53 signaling pathways. Given the size of our dataset, we were unable to draw significant conclusions about certain demographics. Conclusions: This study presents data that can help draw conclusions on common mutations, mutual exclusivity patterns, and demographics at risk for peritoneal mesothelioma. Genomic analysis of peritoneal mesothelioma may inform possible intervention targets for therapeutic treatment.

## 1. Introduction

Malignant peritoneal mesothelioma (MPM) is a rare cancerous tumor originating from the lining of the peritoneum. Mesothelioma is primarily associated with asbestos exposure, though this relationship is notably weaker in peritoneal mesothelioma than in the pleural subtype. Additionally, inherited mutations may contribute to earlier onset in some patients [1]. Pathologically, the disease has been classified into three subtypes: epithelioid, sarcomatoid, and biphasic, each presenting distinct characteristics in terms of appearance, growth, and prognosis [2]. Regarding future care, there are several ongoing clinical trials exploring novel treatment approaches for mesothelioma, including immunotherapy, intraperitoneal chemotherapy, targeted therapies, and surgery, as well as combined treatments when appropriate [3,4,5].

MPM accounts for approximately 10–15% of all mesothelioma cases and represents a clinically distinct subtype [1,6]. In contrast to pleural mesothelioma, peritoneal mesothelioma demonstrates a more balanced sex distribution in reported cohorts [1,7]. Survival outcomes vary widely depending on histologic subtype, tumor burden, and eligibility for cytoreductive surgery with intraperitoneal chemotherapy, with reported median survival ranging from 12 to 92 months [7,8,9]. Known risk factors for developing MPM includes asbestos exposure, a long latency period, and genetic predisposition [1,10,11,12]. Mortality rates vary depending on patient conditions, health factors, and tumor subtypes [13,14,15,16].

Initial evaluation for suspected peritoneal mesothelioma typically includes cross-sectional imaging with CT scans to examine mesothelial linings within the abdominal cavity, along with open biopsy for potential surgical diagnosis and treatment planning [17]. Since mesothelioma can spread diffusely through different surfaces, surgical approaches may not be a practical option for certain patients. Instead, diagnosis and treatment planning rely on laboratory tests, positron emission tomography scans, and radiographic imaging. Disease stage, metastasis, and careful treatment processes based on patient medical history and conditions are considered when making definitive diagnoses and treatment plans [1,17,18]. To classify the tumor, staging systems categorize tumors based on location and subtype. Recent research has found that new investigational therapies, such as targeted therapies, tumor-treating fields, and gene and cell therapies, have been through clinical testing for potential treatments in the future [17,19,20]. Further analysis on mesothelioma pathogenesis is vital for new treatments and diagnoses to help improve emerging treatment strategies to help support the quality of patient and tumor outcomes.

Recent genomic studies have begun to further characterize the molecular landscape of MPM. Investigations have identified recurrent alterations in tumor suppressor genes, including *BAP1*, *NF2*, *CDKN2A*, and other chromatin remodeling genes, suggesting that tumorigenesis is largely driven by inactivation of tumor suppressor pathways rather than by activation of classical oncogenes [3,21,22,23]. Variants in these genes can contribute to differences in tumor behavior, therapeutic response, and clinical outcomes [24,25]. However, many prior genomic analyses have consisted of smaller cohorts or single-institution studies, limiting the ability to comprehensively evaluate mutation co-occurrence patterns and pathway interactions. The current gaps in understanding the molecular and genetic basis of MPM prolong the timeline for diagnosis and treatment, underscoring the need for further investigation using larger multi-institutional datasets.

The objective of this study is to characterize the somatic genomic landscape of MPM using a multi-institutional dataset. By evaluating recurrent mutations, pathway alterations, and mutation co-occurrence patterns, this analysis aims to further define the molecular drivers of MPM and to identify potential targets for therapeutic development. Addressing genomic gaps for MPM can help with targeted therapeutic strategies and a clear understanding of disease pathogenesis. Despite current improvements, a comprehensive understanding of the genetic modifications underlying peritoneal mesothelioma pathogenesis remains incomplete.

## 2. Materials and Methods

Ethical approval was waived by the Creighton University Institutional Review Board as the data used was from the AACR Project GENIE^®^ database (version 17.0-public; American Association for Cancer Research, Philadelphia, PA, USA) and is deidentified. We identified patients with a pathologic diagnosis of peritoneal mesothelioma. The cohort included 192 patients and 204 samples. Inclusion criteria required samples to have valid sequencing data; samples with incomplete data were excluded.

The AACR GENIE^®^ database utilizes a variety of data from 19 international cancer centers, including whole-genome sequencing (WGS), whole-exome sequencing (WES), and targeted gene panels covering 50–555 genes. These different sequencing methods yielded different levels of coverage: 80% of samples were subjected to targeted panel sequencing, which yielded the greatest coverage of over 500×, and 15% were subjected to WES, yielding 150× coverage. The remaining 5% of samples experienced WGS, resulting in approximately 30× coverage. Variant calls included both tumor–normal matched sequencing (approx. 35%) and tumor-only sequencing (approx. 65%). To minimize germline contamination in tumor-only samples, we relied on the GENIE consortium’s filtered mutation data, which excludes common germline variants using dbSNP and the ExAC database. 

Each participating institution utilizes individual programs to identify and label mutations, but all methods fall under GENIE harmonization protocols via the Genome NEXUS. For example, GATK (Broad Institute, Cambridge, MA, USA) is often used for variant detection, and ANNOVAR (Wang Laboratory, University of Pennsylvania, Philadelphia, PA, USA) is then used for annotation. However, each institution may have a different version of this software. In addition, variations can exist within individual institutions, as some data comes from comprehensive sequencing methods such as WGS or WES, while other data comes from target panels looking at up to 555 specific genes. Patient responses to treatment and their clinical outcomes are often included in the database; however, this information is not available for peritoneal mesothelioma, as these details were not recorded. 

Patients with a pathologic diagnosis of peritoneal mesothelioma were identified and sorted as primary (obtained from the site where the cancer began to grow) or metastatic (of a secondary site that spread from the primary tumor). A chi-squared test was then performed on the groups using the proportion of mutated samples in each to compare their rate of mutation frequencies. 

Genomic data (e.g., somatic mutations), histological subtype, and clinical demographics (e.g., age, race, and sex) formed the dataset. Although histologic subtype is listed within GENIE data, it was incomplete and inconsistently reported across contributing institutions. Therefore, histologic subtype genomic analysis was not feasible in this cohort study. Cancer-associated genes essential to the study, such as *KMT2D*, *TP53*, and *PIK3CA*, were included in the majority of panel designs, despite slight differences between institutions. Also consistent across panels was the absence of non-actionable genes and the exclusion of structural variants in the analysis. A greater understanding of genetic drivers or tumor progression, resistance, and metastasis is necessary as comprehensive knowledge of the genetic alterations comprising MPM pathogenesis is incomplete. This study is designed to use publicly available data to characterize the somatic genomic landscape of MPM, ultimately aiming to adequately inform work on future therapeutics and screening methodologies. 

We utilized AACR GENIE v17.0-public. The cohort consisted of 204 samples from 192 patients; where multiple samples existed for a single patient, all were included in the landscape analysis, though 94% of patients contributed a single sample. Tumor Mutational Burden (TMB) scores were extracted directly from the GENIE clinical data files. These scores are generated using the GENIE consortium standardized linear regression models, which normalize TMB calculations across different panel sizes to be comparable to whole-exome sequencing (WES) data. 

Samples were excluded if they lacked valid sequencing data or failed the consortium’s quality control thresholds (e.g., 100× coverage). Analysis was done with R/R Studio (R Foundation for Statistical Computing, Boston, MA, USA). Relationships between categorical variables, including co-occurrence, were analyzed using a chi-squared test, and presented as frequencies and percentages. The *p*-values reported are nominal (two-sided) and exploratory; given the small sample sizes in sub-group analyses, no adjustment for multiple testing was applied. While Fisher’s exact test is often preferred for small counts, chi-squared results are presented here as this test was performed directly in the cBioPortal (Memorial Sloan Kettering Cancer Center, New York, NY, USA) and therefore cannot be changed to other test types.

## 3. Results

### 3.1. Patient Demographics of Peritoneal Mesothelioma

To analyze general demographic trends, primary and metastatic tumor samples were combined due to the limited sample size. Demographic characteristics for 204 tumor samples from 192 patients are summarized in Table 1. In terms of sex, 80 (41.7%) samples were from male patients and 104 (54.2%) from female patients. The sex of the remaining eight (4.17%) patients was unknown. There are no demographic data for age for this cohort, and therefore, age-related risk factors or outcomes could not be evaluated in this cohort study. Regarding ethnicity, 151 patients (78.6%) were classified as non-Spanish/non-Hispanic, 10 (5.2%) as Spanish/Hispanic, and 25 (13.5%) patients had unknown ethnicity. With regard to race, most patients were White (139; 72.4%), followed by Asian (12; 6.3%), Black (8; 4.2%), and other (10; 5.2%). Race information was unknown for 21 (10.9%) patients. Of the tumor samples, 146 (71.6%) were from primary tumors, while 25 (12.3%) samples were from metastatic tumors, and 10 (4.9%) lacked information regarding tumor origin.

### 3.2. Most Prevalent Somatic Mutations and Copy Number Alterations

**Figure 1:**
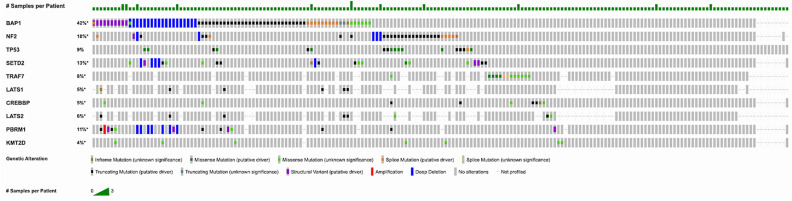
OncoPrint of recurrent mutations in MPM. The differences in sequencing methods yielded different levels of gene coverage, meaning not all genes were evaluated for each sample. Star (*) indicates that not all samples were profiled for that gene. (See Section 2).

The most common mutations found, as shown in Figure 1, were *BAP1* (*n* = 53; 25.98%), *NF2* (*n* = 31; 15.19%), *TP53* (*n* = 19; 9.3%), *SETD2* (*n* = 17; 8.3%), *TRAF7* (*n* = 13; 6.4%), *LATS1* (*n* = 6; 2.9%), *CREBBP* (*n* = 9; 4.4%), *LATS2* (*n* = 8; 3.9%), *PBRM1* (*n* = 8; 3.9%), *KMT2D* (*n* = 7; 2.9%). *TERT* and *SF3B1* also showed significant mutation frequencies, and of this list of genes, the most commonly mutated was *BAP1*. Copy number alterations (CNAs) were also examined. Through this, we found loss-of-heterozygosity (LOH) events were quite prevalent on suppressor genes such as *BAP1* (*n* = 20, 9.8%), *CDKN2A* (*n* = 18, 8.8%), and *CDKN2B* (*n* = 17, 8.3%). In addition, amplifications also showed prevalence, although significantly less than LOH events. These events impact genes such as *WT1* (*n* = 4, 1.96%) and *MCL1* (*n* = 4, 1.96%). Figure 1 demonstrates recurrent mutations in MPM after running the OncoPrint program (Memorial Sloan Kettering Cancer Center, New York, NY, USA).

### 3.3. Genetic Variations by Race and Sex

When grouping cohort data by sex, can be viewed in Table 2, there were only three genes for which differences were nominally significant. Women had the majority of the enrichment of mutations in the *SETD2* (*n* = 20 vs. *n* = 4; *p* = 0.0036) gene, and mutations in the *CDC42* (*n* = 6; *p* = 0.032) gene occurred strictly in women. Given the small subgroup size, this association should be interpreted as exploratory. A mutation in the *SLC45A3* gene (*n* = 1) was identified only in a male patient; however, as this represents a single event, statistical significance cannot be assigned, and this is reported as an observational finding only. In this cohort, there are no statistically significant differences in enrichment of mutations by race. Note that the reported *p*-values are nominal and exploratory, and therefore these findings should be interpreted as hypothesis-generating rather than as confirmatory.

### 3.4. Mutation Co-Occurrence and Mutual Exclusivity Relationships

The most nominally significant co-occurrence patterns were observed between *BAP1* and *PBRM1* (*n* = 19; *p* < 0.001) and *LATS1* and *LATS2* (*n* = 5; *p* < 0.001). Other significant co-occurrence patterns were observed between *BAP1* and *LATS1* (*n* = 6; *p* = 0.013) and *SETD2* (*n* = 17; *p* = 0.003). *LATS1* was also identified with significant co-occurrence patterns with *PBRM1* (*n* = 3; *p* = 0.035). Furthermore, *PBRM1* also had significant co-occurrence with *KMT2D* (*n* = 2; *p* = 0.014). Only one significant mutual exclusivity pattern was identified between *BAP1* and *TRAF7* (*n* = 0, *p* < 0.001).

### 3.5. Primary and Metastatic Mutations

Comparisons between primary and metastatic groups were performed on a per-sample basis. Of the nominally significant results, the majority showed mutations found on genes only in metastatic tumors. This group includes mutations found in *CHEK2* (*n* = 2; *p* = 0.0187), *SRSF2* (*n* = 2; *p* = 0.0188), and *CARM1* (*n* = 2, *p* = 0.0303). There was one instance of a mutation being found on a gene only in primary tumor samples, which was in the *TPM1* (*n* = 2, *p* = 0.0230). In *PBRM1* (*n* = 6 vs. *n* = 12; *p* = 0.0262), there were more instances of mutation in patients with primary tumors; however, due to the difference in group sizes, a much greater percentage of metastatic tumors displayed a mutation on this gene. There were no genes with similar numbers of mutation enrichment.

## 4. Discussion

### 4.1. Study Overview and Key Findings

This study analyzed data on peritoneal mesothelioma through the American Association for Cancer Research (AACR) Project Genomics Evidence Neoplasia Information Exchange (GENIE) database. Analysis of this data revealed recurrent mutations in key tumor suppressor genes. Among the tumor suppressor genes analyzed, *BAP1*, *NF2*, and *TP53* had the most frequent mutations. Regarding sex-specific differences in mutated genes, women had significant enrichment in the *SETD2* gene. Mutations in *CDC42* only occurred in female patients. Mutations in the *SLC45A3* gene were only observed in male patients. Based on co-occurrence analysis, *BAP1* and *PBRM1* is a notable pattern pairing as is *CHEK2* and *SRSF2* when comparing primary and metastatic samples. In this observed dataset, there were no race-specific differences in mutation enrichment.

### 4.2. Demographic Differences and Mutation Patterns

The observed cohort consisted of 41.7% (*n* = 80) male and 54.2% (*n* = 104) female patients. Notably, female patients had enrichment in the *SETD2* gene (female: *n* = 20, male: *n* = 4, *p* = 0.0036). In addition, mutation in the *CDC42* gene was only observed in female patients (female: *n* = 6, male *n* = 0, *p* = 0.032). Mutation in the *CDC42* gene found exclusively in women suggests that there is a possible sex-linked biological impact that requires further investigation. Historically, according to the literature, females with mesothelioma had improved overall survival compared with males [7,9]. While survival data was unavailable within the GENIE dataset, the enrichment of *SETD2* and *CDC42* mutations particularly in females is of note. These mutations may correlate with the better overall prognosis typically observed in female mesothelioma patients and more specifically in malignant peritoneal mesothelioma female patients. Sex-based biological differences could contribute to the enrichment of both *SETD2* and *CDC42* mutations. Additionally, the *SLC45A3* mutation was exclusive to males (male: 1, female: 0, *p* = 0.027), although only one case was observed.

Ethnically, this cohort was predominantly non-Spanish/non-Hispanic (*n* = 151, 78.6%). In addition, a majority of the patients in the cohort identified as White (*n* = 139, 72.4%). This is consistent with studies that indicate there is a higher incidence of peritoneal mesothelioma in non-Hispanic White patients [1]. According to the literature, in the United States, approximately 90% of new peritoneal mesothelioma cases are diagnosed in non-Hispanic White patients [26]. Understanding that peritoneal mesothelioma has a higher incidence rate among non-Hispanic White patients can provide insight into potential contributing factors to the cancer. However, in this observed dataset, there were no race-specific statistically significant differences in mutation enrichment. Given the relatively low number of non-White patients in this dataset (Asian: *n* = 12, Black: *n* = 8, other: *n* = 10, unknown: *n* = 21), a larger, more-representative cohort is needed to draw significant conclusions about race-specific differences in mutations. 

### 4.3. Commonly Mutated Genes and Altered Pathways

Consistent with the previous literature, our peritoneal mesothelioma cohort exhibited significant mutation within the *BAP1* gene (25.98%) [1,6,27]. This study also identified common somatic mutations in *NF2* (15.19%), *TP53* (9.3%) and *SETD2* (8.3%). Mutations were also observed in the *TRAF7* (6.4%), *LATS1* (2.9%), *CREBBP* (4.4%), *LATS2* (3.9%), *PBRM1* (3.9%), *KMT2D* (2.9%) genes, but to a lesser extent. These findings align with prior studies that have determined that the loss or mutation of the *BAP1* gene is linked to peritoneal mesothelioma [1,6,27,28]. *BAP1* is associated with tumor suppressor genes and is related to DNA damage repair pathways [6,27,29]. Another common altered pathway, *NF2*, is associated with the Hippo pathway, which is a key component in the regulation of cell proliferation [6,27,30].

Through examination of CNAs, LOH events were predominant in *BAP1* (9.8%), *CDKN2A* (8.8%), and *CDKN2B* (8.3%) and less predominant in *WT1* (1.96%) and *MCL1* (1.96%). Along with common mutations within *BAP1*, *NF2*, and *TP53*, mutation of the *CDKN2A/B* gene is frequently associated with peritoneal mesothelioma [6,29].

The clinical standard for treatment of peritoneal mesothelioma can be attributed to its interaction with these commonly mutated pathways associated with the mutation of *BAP1*, *NF2*, *TP53*, and *CDKN2A/B* genes. While recurrent mutations in these genes can highlight biologically relevant pathways, mutation frequency alone does not establish therapeutic actionability. Therefore, references to “potential therapeutic targets” in this manuscript indicate biologically plausible hypotheses rather than clinically validated interventions specific to peritoneal mesothelioma. Results from our study and the previous literature suggest that peritoneal mesothelioma tumorigenesis is driven by the deactivation of tumor suppressor genes rather than by the activation of oncogenes [1,6,27,28,29,30,31]. 

Recent studies have shown that tumorigenesis is driven more by the disruption of tumor suppressor pathways and chromatin regulatory complexes [32,33,34]. Analyses done by Duan et al., Li et al., and Sun et al. have demonstrated that alterations in the *BAP1*, *NF2*, and *SETD2* genes are likely part of a combination of drivers in tumorigenesis. Rather than a singular oncogene activation, these studies have shown that there is a more coordinated inactivation of tumor suppressor and chromatin regulatory networks leading to tumorigenesis [32,33,34]. These comprehensive sequencing studies reinforce the concept that peritoneal mesothelioma could be characterized by a network of tumor suppressors [6,30,35].

Beyond descriptive mutation frequency, studies have demonstrated that recurrent inactivation of tumor suppressor genes such as *BAP1*, *NF2*, and *CDKN2A/B* is strongly associated with cancer risk and progression [27,29,32,36]. Recurrent alterations in tumor suppression genes have been biologically linked to differences in tumor behavior and potential therapeutic responses [27,37,38]. Findings from Ning et al. provide a translational framework for the recurrent inactivation of *BAP1*, *NF2*, *TP53*, and *CDKN2A/B* seen in our cohort [37]. Ultimately, mutations in the tumor suppressor genes in peritoneal mesothelioma are biologically significant rather than coincidental.

### 4.4. BAP1 and Chromatin Remodeling

The most frequently mutated gene found in our cohort was *BAP1* (*n* = 53, 25.98%). This frequency is consistent with previous genomic studies, such as Singhi et al. (2016) and Hiltbrunner et al. (2022), which reported *BAP1* mutation rates of approximately 23% to 30% in smaller, single-institution cohorts [27,35]. This is consistent with previous studies, one of which found a very similar 23% *BAP1* mutation among their cohort [38]. Other studies have found that *BAP1* loss is often a common defining genomic feature in mesotheliomas [38]. *BAP1* ensures genomic stability and efficient DNA replication and therefore suppresses cancer development [29,39,40,41]. Since *BAP1* has been identified as a key tumor suppressor, it is a hopeful potential therapeutic target but has been found to be a complex target that is resistant to many current forms of therapy [29]. Clinical trials are currently studying the role of *BAP1* [42,43]. Loss of *BAP1* impairs homologous recombination repair, making tumors more susceptible to PARP inhibitors [44]. *BAP1* deficiency also leads to the upregulation of *EZH2*, providing a potential target for therapeutic methods. *EZH2* inhibitors are currently being investigated for *BAP1*-deficient cases of mesothelioma [45]. The *EZH2* inhibitor tazemetostat was tested in a clinical trial, NCT02860286 for its efficacy [44,45,46]. The trial results found that at 12 weeks there was a 54% disease control rate. However, no complete responses were observed in the trial [46]. In preclinical models, *BAP1* deficiency also leads to upregulation of *EZH2*, histone deacetylases (HDACs), and immune checkpoint molecules such as *PD-1*, *PD-L1*, and *LAG3*, providing multiple potential therapeutic targets. The *EZH2* inhibitor tazemetostat is currently being investigated in *BAP1*-inactivated mesothelioma (e.g., NCT03830229) [42]. While early-phase studies showed a disease control rate of 54% at 12 weeks, complete responses were not observed, suggesting that biomarkers beyond just *BAP1* may need to be explored for therapeutic treatment [45]. Many of the therapeutic treatment strategies involving *BAP1* remain largely preclinical, but some provide future directions that may have promising results [44].

Importantly, most clinical trials evaluating targeted genomic pathways in mesothelioma have primarily enrolled patients with malignant pleural mesothelioma, with peritoneal cases either being underrepresented in the trial or not separately reported. Clinical trial, NCT02860286, was conducted in pleural mesothelioma patients with *BAP1* inactivation, and peritoneal-mesothelioma-specific outcomes were not reported separately [46].

### 4.5. Hippo Signaling and NF2 Mutation

*NF2* gene mutations were the second most common in our cohort, with 15.19% of patients presenting *NF2* mutations. Similarly, another study found 19% of patients with the *NF2* gene mutation in another sample [38]. *NF2* regulates the downstream Hippo pathway via association of *LATS1/LATS2,* which causes the suppression of *YAP* and *TAZ*, which are proteins associated with cell growth [6,27,30]. Clinically, *YAP/TAZ* activation is linked to poorer outcomes in mesothelioma patients, making it a potential option for targeted therapy [47]. The mutation of *NF2* can lead to the hyperactivation of *YAP* and therefore leads to uncontrollable cell proliferation and tumor growth [30,35,48,49]. Beyond *NF2′s* role in *LATS1/LATS2*, *NF2* may also directly interact with transcription factor *TEAD4*, which is also involved in the Hippo signaling pathway [49]. This direct association can offer an alternative route to Hippo signaling deregulation in peritoneal mesothelioma.

*YAP*, *TAZ*, and *TEAD4* inhibitors such as IAG933 are in phase I clinical trials for mesothelioma patients [49,50]. This trial is investigating the efficacy of disrupting the *YAP* pathway and treating the Hippo signaling pathway [51]. Additionally, early-phase studies of YAP, TAZ, and TEAD inhibitors have predominantly enrolled pleural mesothelioma and solid tumor cohorts [50]. Ultimately, there need to be dedicated prospective trials in peritoneal mesothelioma to validate whether pathway dependencies are conserved across mutated gene sites. However, focusing on the targeting of the *NF2* gene mutations and Hippo pathway could be an effective way of treating and slowing the progression of peritoneal mesothelioma [30,51].

### 4.6. p53 Pathway

The third most common mutation in our cohort occurred in the *TP53* gene with 9.3% of the patients presenting a mutation. This is consistent with the other literature results that found the mutation rate for *TP53* ranges from around 8 to 14.6% [35,38]. Loss of *TP53*, and therefore p53, function disrupts cell cycle regulation and leads to genomic instability and uncontrolled cell proliferation and tumorigenesis [38]. In mesothelioma, *TP53* is not the only mutation that can disrupt the p53 pathway. Alterations in checkpoint kinases, like *CHEK2*, and negative regulators, such as MDM2, can alter the p53 pathway [52,53]. In our cohort, *CHEK2* mutations were observed in metastatic tumors, which is consistent with the literature that states *CHEK2* and *TP53* mutations have critical roles in tumor suppression. In other types of cancer, *CHEK2* and *TP53* mutations have linked expression patterns and frequently co-occur, which reinforces that disruption in the p53 pathway can alter DNA damage response [53]. For example, in a study, *CHEK2* and p53 status was explored in 196 gastric cancer patients [52]. Results found that *CHEK2* mutations and p53 status are correlated, ultimately suggesting that there is an interplay between the two DNA damage response components [52]. So, in mesothelioma, damage or loss of the p53 pathway can arise from direct mutations of *TP53*, mutations in checkpoint kinases (i.e., *CHEK2*), or the amplification or overexpression of p53 negative regulators (i.e., MDM2).

Regarding potential therapeutic interventions for *TP53* mutations, MDM2 inhibitors can restabilize the p53 pathway. For *TP53* wildtype tumors, MDM2 inhibitors can stabilize and reactivate p53. In a clinical trial NCT01877382, an MDM2 inhibitor called milademetan has been explored, and results show that inhibition of MDM2 could be a possible method of restabilizing the p53 pathways and suppressing tumor growth activity [52]. In addition, WEE1 inhibition is being clinically researched in clinical trials NCT01164995 and NCT02448329, in p53-mutated cells [54,55,56]. Clinical trial results from NCT01357161 have shown that adavosertib, a WEE1 inhibitor, has anti-tumor activity in TP53-mutated tumors [36,57,58,59]. These trials have predominately enrolled patients without peritoneal mesothelioma, making it difficult to draw significant conclusions related to intervention possibilities. But these observations in the trials highlight the p53 pathway as a potential critical component of mesothelioma biology. The p53 pathway should be further clinically explored as a potential therapeutic target for peritoneal mesothelioma. 

### 4.7. Co-Occurrence and Mutual Exclusivity Patterns

The analysis of our cohort presented several patterns in gene co-occurrence. There were statistically significant results between the *BAP1* and *PBRM1* (*n* = 19; *p* < 0.001) and *LATS1* and *LATS2* (*n* = 5; *p* < 0.001) genes. *BAP1* and *PBRM1* are associated with chromatin remodeling and DNA damage repair, and the mutation of these genes concurrently could disrupt proper gene expression [35]. We also observed significant co-occurrence with the *LATS1* and *LATS2* (*n* = 5; *p* < 0.001) genes. *LATS1/LATS2*, tumor suppressor kinases, both have significant roles in the Hippo signaling pathway [30,48,49,51]. In the Hippo signaling pathway, *NF2* activates *LATS1/LATS2,* which then phosphorylates and deactivates *YAP* and TEADs, leading to uncontrolled cell proliferation and tumorigenesis [30,49,51]. However, in contrast, new results have also found that *NF2* can also directly interact with *TEAD4*, rather than via *LATS1/LATS2* and *YAP* [49]. The literature found that *NF2* and *TEAD4* directly interact via *NF2* interacting with the FERM domain and C-terminal tail of *TEAD4* [49]. This means that *NF2* can decrease the stability of *TEAD4* without interacting with *LATS1/LATS2* and *YAP* [49]. Therefore, lack of *NF2* can stabilize *TEAD4* even if the *LATS1/LATS2* and *YAP* portion of the pathway is still intact [49]. Thus, the *LATS1/LATS2* co-occurrence may suggest that, in order to uphold TEAD activity, there is genomic selection for loss of *LATS* kinase function. Due to our sample size being small, further research would need to be conducted to support the conclusion that either *NF2* loss or *LATS1/2* loss can lead to TEAD activation [49,51].

The dual deactivation of *LATS1/LATS2* likely still disrupts the Hippo signaling pathway and promotes uncontrolled cell proliferation and tumorigenesis. In addition, co-occurrence was observed with *BAP1* and *LATS1* (*n* = 6; *p* = 0.013) and *SETD2* (*n* = 17; *p* = 0.003). *LATS1* and *PBRM1* (*n* = 3; *p* = 0.035) and *PBRM1* and *KMT2D* (*n* = 2; *p* = 0.014) genes also had a statistically significant co-occurrence pattern. These patterns suggest a cooperative role in tumorigenesis and mesothelioma growth [21].

In our cohort, we observed only one statistically significant mutually exclusive co-occurrence between *BAP1* and *TRAF7* (*n* = 0; *p* < 0.001). Since these genes are mutually exclusive, it is likely that these genes were never mutated in the same tumor concurrently. *BAP1* mutations are frequently associated with chromatin pathway disruption and malignant forms of peritoneal mesothelioma [22,28,29]. *TRAF7* is a component in a separate pathway than the pathway *BAP1* uses, suggesting why it may be mutually exclusive to *BAP1*. *BAP1* and *TRAF7* being mutually exclusive suggests that there are alternative mutations, such as in *TRAF7*, to promote the cell proliferation and tumorigenesis in mesothelioma [60]. These mutually exclusive patterns within the chromatin remodeling and Hippo pathways could be used to inform future treatment and targeted therapeutic strategies.

### 4.8. Primary vs. Metastatic Mutation Patterns

In our cohort study, we also compared and looked at patterns between primary and metastatic mutations. Of all the statistically significant results, many of the gene mutations were found in metastatic tumors. Mutations in the following genes were only found in metastatic tumors: *CHEK2* (*n* = 2; *p* = 0.0187), *SRSF2* (*n* = 2; *p* = 0.0188), and *CARM1* (*n* = 2, *p* = 0.0303). Mutations within the *PBRM1* gene were observed in both metastatic and primary tumors. *PBRM1* mutations were numerically more common in primary tumors (*n* = 12) than in metastatic tumors (*n* = 6). Although, the smaller size of the metastatic group meant that a larger proportion of the metastatic tumors in our cohort carried *PBRM1* mutations in total. These patterns suggest that the enrichment of *CHEK2*, *SRSF2*, and *CARM1* may occur after or during metastasis. While the enrichment of *PBRM1* may occur prior to metastasis. These enriched genes all play a role in tumor progression. *CHEK2*, a tumor suppressor gene, is relevant in cell cycle regulation, and the loss of its function can lead to improper cell proliferation [23]. *SRSF2* is involved in splicing events, and enrichment of this gene may lead to altered RNA processing [61]. *CARM1* is an arginine methyltransferase protein that is responsible for steps within the RNA production process [62]. In addition to the mutations found in metastatic tumors, we also observed a mutation in *TPM1* (*n* = 2, *p* = 0.0230) only in primary tumors. The association of these mutations with metastatic tumors presents the possibility that they are needed or contribute to the dissemination capacity of the peritoneal tumor cells, although these associations require further validation and study.

However, given the imbalance of primary (*n* = 146) and metastatic (*n* = 25) samples, this analysis is vulnerable to sampling noise. Observed metastatic-exclusive mutations (e.g., *CHEK2*, *SRSF2*, *CARM1*) may have limited statistical power rather than presenting true biological selection. Therefore, these findings should be interpreted cautiously and validated with further study of datasets.

### 4.9. Study Limitations

This study has several limitations inherent to the AACR GENIE dataset. First, the dataset lacks clinical outcome data, such as overall survival and treatment history, preventing analysis of the prognostic impact of the identified mutations. Second, the database aggregates data from multiple institutions using different sequencing panels with varying gene coverage (heterogeneity), which may affect the reported mutation frequencies. Third, while this cohort (*n* = 192) is large for this rare disease, the sample size remains too small to conduct robust stratified analyses by race or histological subtype (e.g., epithelioid vs. sarcomatoid), as these subtypes are often aggregated in the database. Finally, this is a descriptive genomic landscape analysis; no functional (wet-lab) validation of the mutations or pathway interactions was performed. Inability to track tumor evolution over time or compare primary and metastatic tumors from patients proved to be another limiting factor. There is also a possibility that the dataset used contains multiple tumor samples from the same patient, although prior evaluations suggest that this would have minimal effect on overall trends seen in the data. Since we used genomic data, we cannot evaluate and capture non-genetic driving forces, such as microenvironmental factors, in the progression of peritoneal mesothelioma. There was also a lack of transcriptomic and miRNA data in the GENIE dataset. Without transcriptomic data, we were unable to link downstream pathway activation to mutation status or actual gene expression. Similarly, our ability to explore epigenetic regulation in tumor progression and initiation was limited due to the fact that no DNA methylation data was available to us during this study. Additionally, the GENIE database groups all histologic subtypes of peritoneal mesothelioma (epithelioid, sarcomatoid, and biphasic) together. Without specific subtype data, we could not analyze certain genomic alterations and enrichments that are found in particular morphologic variants. There was also a lack of immunohistochemistry data. This meant we could not examine how genetic changes translate into differences in protein expression. Taking these constraints into account, our analysis can offer insight to mutations in pathways such as the *BAP1* and chromatin, Hippo signaling, and p53 pathways. However, note that the frequencies of mutations in genes such as *BAP1* are slightly inflated due to unfiltered germline variants represented in the tumor-only cohort. These results can help us characterize and understand peritoneal mesothelioma, hopefully laying the groundwork for future studies and clinical research for new therapeutic interventions.

Additionally, the data originated from sequencing panels of varying sizes and gene content. While GENIE harmonizes these data, incomplete coverage of certain genes across different panels is a limitation. The commonly applied filtering criteria (VAF ≥ 5%, coverage ≥ 100×) utilized to ensure high-confidence calls may result in the exclusion of sub-clonal driver mutations. Furthermore, as a significant portion of samples underwent tumor-only sequencing, residual germline contamination remains a possibility despite filtering. We utilized the native statistical tools provided by the GENIE/cBioPortal consortium to ensure our methodology aligns with platform defined outputs from this platform. We have acknowledged the limitations of using chi-squared for smaller subgroups in the text and urge interpretation of these *p*-values as exploratory. We acknowledge that the use of chi-squared testing in small subgroups may be less robust than Fisher’s exact testing, and therefore these *p*-values should be interpreted as nominal, exploratory, and unadjusted and therefore should be viewed as hypothesis-generating.

## 5. Conclusions

This study provides a comprehensive analysis of the genomic mutational landscape of peritoneal mesothelioma using the AACR Project GENIE database. Recurrent genomic alterations were identified in *BAP1*, *NF2*, *TP53*, and *SETD2*. In addition, there were potential therapeutic targets within the chromatin remodeling, Hippo signaling, and p53 pathways. Demographic patterns, co-occurrence, and mutually exclusive patters were analyzed and observed, highlighting distinct genomic patterns. The findings expand the current genomic insight into peritoneal mesothelioma, which may guide future research, clinical trial design, and targeted therapeutic intervention.

## Figures and Tables

**Table 1:** Patient demographics for peritoneal mesothelioma.

Demographics	Category	*n* (%)
Sex	Male	80 (41.7)
	Female	104 (54.2)
Ethnicity	Non-Hispanic	151 (78.6)
	Unknown/Not collected	25 (13.5)
	Hispanic	10 (5.2)
Race	White	139 (72.4)
	Asian	12 (6.3)
	Black	8 (4.2)
	Other	10 (5.12)
	Unknown	21 (10.9)
Sample type	Primary	146 (71.6)
	Metastasis	25 (12.3)
	Not collected	10 (4.9)

**Table 2:** Sex and associated mutations in patients diagnosed with mesothelioma.

Gene (Chi-Squared)	Male, *n* (%)	Female, *n* (%)	*p* Value
*SETD2*	4 (4.76)	20 (19.42)	*p* = 0.0036
*CDC42*	0 (0.0)	6 (10.34)	*p* = 0.032
*SLC45A3*	1 (33.33)	0 (0.0)	n/a

## Data Availability

The data presented in this study are available from the AACR GENIE database at https://genie.cbioportal.org/ (accessed on 6 February 2025).

## Abbreviations

The following abbreviations are used in this manuscript:

AACR	American Association for Cancer Research
ASR	Age-Standardized Rate
BAP1	BRCA1-Associated Protein 1
CARM1	Coactivator-Associated Arginine Methyltransferase 1
CDKN2A	Cyclin-Dependent Kinase Inhibitor 2A
CDKN2B	Cyclin-Dependent Kinase Inhibitor 2B
CHEK2	Checkpoint Kinase 2
CNAs	Copy Number Alterations
CT	Computed Tomography
DNA	Deoxyribonucleic Acid
EZH2	Enhancer of Zeste Homolog 2
FDR	False Discovery Rate
GENIE	Genomics Evidence Neoplasia Information Exchange
HDAC	Histone Deacetylase
H2A-Ub	Monoubiquitinated Histone H2A
LATS1	Large Tumor Suppressor 1
LATS2	Large Tumor Suppressor 2
LOH	Loss of Heterozygosity
MCL1	Myeloid Cell Leukemia 1
MDM2	Mouse Double Minute 2 Homolog
MPM	Malignant Peritoneal Mesothelioma
NF2	Neurofibromatosis Type 2
PARP	Poly (ADP-ribose) Polymerase
PBRM1	PolyBromo 1
SETD2	SET Domain Containing 2
SF3B1	Splicing Factor 3B Subunit 1
SRSF2	Serine- and Arginine-Rich Splicing Factor 2
TAZ	Tafazzin
TEAD4	TEA Domain Transcription Factor 4
TERT	Telomerase Reverse Transcriptase
TMB	Tumor Mutational Burden
TPM1	Tropomyosin 1
TP53	Tumor Protein p53
TRAF7	TNF Receptor-Associated Factor 7
KMT2D	Histone-Lysine N-Methyltransferase 2D
WEE1	WEE1 G2 Checkpoint Kinase
WES	Whole-Exome Sequencing
WGS	Whole-Genome Sequencing
WT1	Wilms’ Tumor 1
YAP	Yes-Associated Protein
